# MLK3 regulates FRA-1 and MMPs to drive invasion and transendothelial migration in triple-negative breast cancer cells

**DOI:** 10.1038/oncsis.2017.44

**Published:** 2017-06-12

**Authors:** C Rattanasinchai, B J Llewellyn, S E Conrad, K A Gallo

**Affiliations:** 1 Department of Physiology, Michigan State University, East Lansing, MI, USA; 2 Cell and Molecular Biology Program, Michigan State University, East Lansing, MI, USA; 3 Department of Microbiology and Molecular Genetics, Michigan State University, East Lansing, MI, USA

## Abstract

Mixed-lineage kinase 3 (MLK3), a mitogen-activated protein kinase kinase kinase (MAP3K), has critical roles in metastasis of triple-negative breast cancer (TNBC), in part by regulating paxillin phosphorylation and focal adhesion turnover. However the mechanisms and the distinct step(s) of the metastatic processes through which MLK3 exerts its influence are not fully understood. Here we report that in non-metastatic, estrogen receptor-positive breast cancer (ER+ BC) cells, induced MLK3 expression robustly upregulates the oncogenic transcription factor, FOS-related antigen-1 (FRA-1), which is accompanied by elevation of matrix metalloproteinases (MMPs), MMP-1 and MMP-9. MLK3-induced ER+ BC cell invasion is abrogated by FRA-1 silencing, demonstrating that MLK3 drives invasion through FRA-1. Conversely, in metastatic TNBC models, high FRA-1 levels are significantly reduced upon depletion of MLK3 by either gene silencing or by the CRISPR/Cas9n editing approach. Furthermore, ablation of MLK3 or MLK inhibitor treatment decreases expression of both MMP-1 and MMP-9. Consistent with the role of tumor cell-derived MMP-1 in endothelial permeability and transendothelial migration, both of these are reduced in MLK3-depleted TNBC cells. In addition, MLK inhibitor treatment or MLK3 depletion, which downregulates MMP-9 expression, renders TNBC cells defective in Matrigel invasion. Furthermore, circulating tumor cells derived from TNBC-bearing mice display increased levels of FRA-1 and MMP-1 compared with parental cells, supporting a role for the MLK3–FRA-1–MMP-1 signaling axis in vascular intravasation. Our results demonstrating the requirement for MLK3 in controlling the FRA-1/MMPs axis suggest that MLK3 is a promising therapeutic target for treatment of TNBC.

## Introduction

Metastatic breast cancer is responsible for nearly half a million deaths worldwide^1^ and 40 000 deaths in the United States,^2^ annually. A major contributor is a lack of efficacy of the current standard treatments in preventing and treating metastatic disease. Breast cancer metastasis is a multistep process initiated by cancer cells within a primary tumor that gain invasive capacity. These cancer cells must breach the basement membrane, invade through extracellular matrix and intravasate into blood vessels. The cells that intravasate into the bloodstream, circulating tumor cells (CTCs), must survive in the circulation, extravasate to a distant site and colonize to form metastatic lesions.^3^ Of the major clinical breast cancer subtypes, triple-negative breast cancer (TNBC) is considered the most aggressive and has the highest rate of metastasis and early recurrence.^4^ Given the relative dearth of targeted therapies for treating TNBC, standard treatment relies on surgical removal, adjuvant radiotherapy and toxic chemotherapy.

Mixed-lineage kinase 3 (MLK3) is a mitogen-activated protein kinase kinase kinase (MAP3K) that transduces signals from multiple cell surface receptors to activate MAPK cascades in a context-dependent manner.^5, 6^ Activated MAPKs directly phosphorylate cytosolic substrates or undergo nuclear translocation to regulate transcription factors, including activating protein-1 (AP-1).^5, 6^ MLK3 is critical for TNBC metastasis.^7, 8^ We have shown in TNBC models that MLK3 mediates JNK-dependent paxillin phosphorylation to facilitate focal adhesion turnover and cell migration.^8^ In addition, MLK3 signaling leads to JNK-mediated c-JUN phosphorylation,^9^ which activates AP-1-mediated gene expression.

AP-1 transcription factors comprise, usually heterodimeric, combinations of JUN and FOS family members including c-JUN, JUN-B, JUN-D, c-FOS, FOS-B, FRA-1 and FRA-2.^10^ Aberrant AP-1 activity regulates genes that promote cancer progression.^11, 12^ Among AP-1 members, high levels of FRA-1 are associated with poor prognosis in TNBC.^11, 13^ FRA-1 is elevated in TNBC cell lines compared with estrogen receptor-positive breast cancer (ER+ BC) cell lines;^14, 15^ and is required for proliferation,^15^ epithelial-to-mesenchymal transition,^13, 16^ invasion^17, 18^ and metastasis.^19^ Invasion genes controlled by FRA-1 include matrix metalloproteinases (MMPs), the zinc-dependent endopeptidases involved in matrix degradation and extracellular matrix remodeling.^20^ Elevated levels of several MMPs are found in many types of solid tumors; these MMPs have crucial roles in multiple steps of tumor progression including tumor growth, angiogenesis, invasion and metastasis.^21^

In this study, we demonstrate that MLK3 is a key regulator of FRA-1 expression in both ER+ BC and TNBC models. Furthermore, we show that the MLK3–FRA-1 axis controls levels of MMP-1 and MMP-9. Consistent with the roles of these MMPs, loss of MLK3 blocks Matrigel invasion as well as transendothelial migration of highly aggressive 4T1 cells. Importantly, an MLK inhibitor diminishes FRA-1 and its target genes, MMP-1 and MMP-9, in TNBC cells suggesting that targeting MLK3 may interfere with metastatic progression.

## Results

### MLK3 is required for FRA-1 expression in breast cancer cells

**Figure 1:**
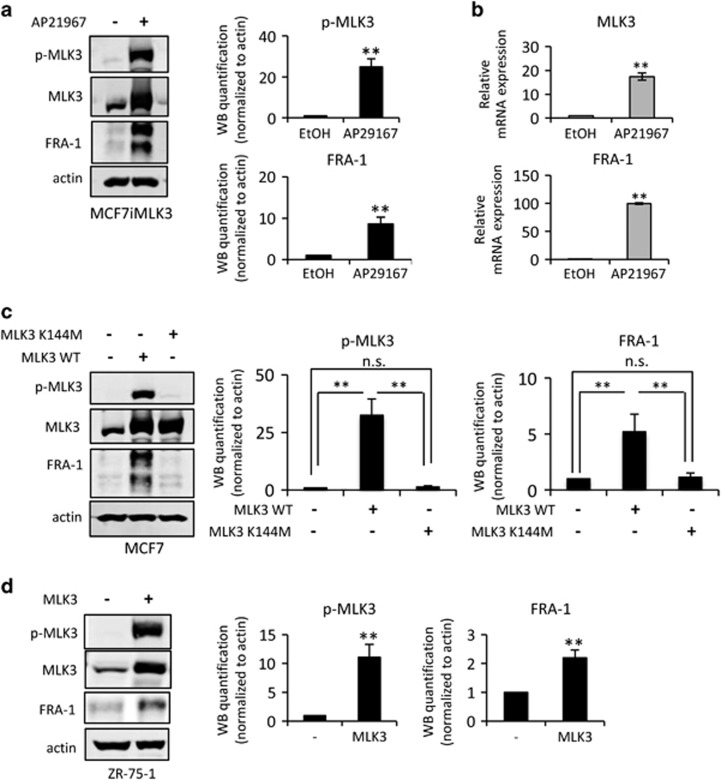
Ectopic expression of MLK3 drives FRA-1 expression in ER+ BC cells. Cellular lysates and/or mRNAs were collected from (**a** and **b**) MCF7iMLK3 cells treated with vehicle or 50 nm AP21967 to induce MLK3 expression for 24 h, (**c**) MCF7 cells were transiently transfected with a wild-type MLK3 (pRK-MLK3) or a kinase dead MLK3 variant (pRK-MLK3-K144M) for 24 h, and (**d**) ZR-75-1 cells transiently transfected with pRK-MLK3 expression vector for 24 h. Cellular lysates were subjected to immunoblotting with indicated antibodies. Western blot quantification of the indicated protein normalized to actin is expressed as mean±s.d. from at least three independent experiments. The mRNAs were subjected to qRT–PCR with primers for the indicated genes. Relative mRNA expression is displayed as mean±s.d. from at least three independent experiments performed in triplicate; NS, not statistically significant; **P<0.01.

High FRA-1 levels are found in aggressive TNBC, whereas ER+ BC cell lines typically have low FRA-1 levels and are poorly invasive.^14, 15^ To examine whether MLK3 promotes FRA-1 expression in ER+ BC cells, we utilized MCF7 cells engineered to overexpress MLK3 upon treatment with the transcriptional inducer AP21967 (MCF7iMLK3).^9, 22, 23^ As shown in Figure 1a, induced MLK3 expression increases FRA-1 protein expression, and drives cell migration in both transwell^9^ and wound-healing assays (Supplementary Figure 1). As FRA-1 is an AP-1-regulated gene,^24^ and MLK3 is known to activate AP-1,^9^ quantitative reverse transcribed PCR (qRT–PCR) analysis was performed to determine FRA-1 transcript levels. As shown in Figure 1b, MLK3 robustly increases the FRA-1 transcript level. Consistent with our previous findings,^22^ ectopically expressed MLK3 is active as judged by phospho-MLK3 immunoblotting (Figure 1a). To assess whether MLK3 catalytic activity is required for FRA-1 expression, vectors encoding wild-type MLK3 or a kinase inactive mutant MLK3 K144M^23^ were transiently introduced into MCF7 cells. Wild-type MLK3 drives FRA-1 expression, whereas ectopic expression of equivalent protein levels of MLK3 K144M fails to upregulate FRA-1 (Figure 1c). As shown in Figure 1d, overexpression of wild-type, active MLK3 in ER+ ZR-75-1 cells also drives FRA-1 expression. Thus, high levels of active MLK3 can upregulate FRA-1 expression in multiple ER+ BC cells.

**Figure 2:**
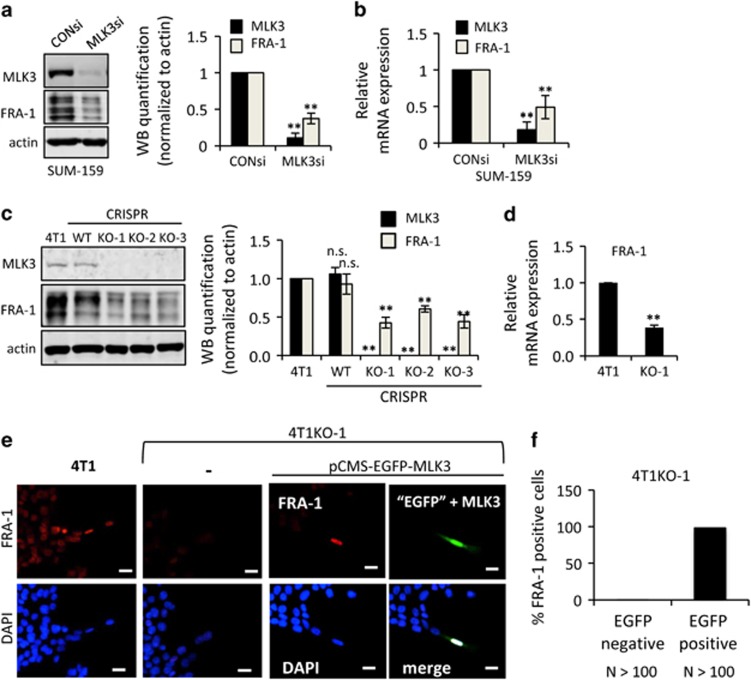
MLK3 is required for FRA-1 expression in TNBC cells. Cellular lysates and/or mRNA samples were collected from (**a** and **b**) SUM-159 cells treated with 50 nm control siRNA or MLK3 siRNA for 24 h, (**c**) parental 4T1, WT clone and three 4T1 CRISPR MLK3-knockout clones (KO-1, KO-2 and KO-3) and (**d**) parental 4T1 or 4T1KO-1 cells. Cellular lysates were subjected to immunoblotting with indicated antibodies. Western blot quantification of the indicated protein normalized to actin is expressed as mean±s.d. from at least three independent experiments. The mRNAs were subjected to qRT–PCR with primers for the indicated genes. Relative mRNA expression is displayed as mean±s.d. from at least three independent experiments performed in triplicate. (**e** and **f**) Parental 4T1 cells or 4T1KO-1 cells were transfected with bi-cistronic vector expressing EGFP and MLK3 (pCMS-EGFP-MLK3) for 24–48 h and were subjected to immunofluorescence staining using a FRA-1 antibody. FRA-1 staining is shown in red and GFP, which indicates co-expression of MLK3, is shown in green. Nuclei were counterstained with DAPI (blue); Scale bar, 25 μm; NS, not statistically significant; **P<0.01.

In complementary experiments, we investigated the requirement for MLK3 in metastatic TNBC cell lines, which possess high endogenous levels of FRA-1.^15^ MLK3 silencing in highly invasive TNBC SUM-159 cells reduces both FRA-1 protein (Figure 2a) and mRNA (Figure 2b) levels, compared with SUM-159 cells transfected with control siRNA. To evaluate the function of MLK3 in the highly metastatic murine TNBC 4T1 model, we first generated MLK3 gene knockout 4T1-luc2 cells using the CRISPR/Cas9n (nickase)^25^ system (Supplementary Figure 2A). Three MLK3-knockout (MLK3 KO) clones, 4T1KO-1, 4T1KO-2 and 4T1KO-3, as well as a wild-type (WT) clone that maintained MLK3 expression, were confirmed by sequencing (Supplementary Figure 2B). All three 4T1KO clones lack MLK3 expression and show decreased FRA-1 protein expression in contrast to parental 4T1 cells and the WT clone (Figure 2c). Based on qRT–PCR analysis, FRA-1 mRNA transcripts are also reduced in MLK3 KO 4T1 cells compared with parental cells (Figure 2d).

Nuclear FRA-1 expression, observed in parental 4T1 cells, is nearly absent in the 4T1KO-1 cells (Figure 2c). To validate the specificity of CRISPR MLK3 KO, a rescue experiment was performed by transiently transfecting a bi-cistronic pCMS-EGFP-MLK3 vector. EGFP-positive cells, which coexpress MLK3, regain FRA-1 expression (Figure 4e, Supplementary Figure 2C). Because 4T1 cells are poorly transfectable, multiple slides were used to score 100 EGFP-positive cells for FRA-1 expression. As shown in Figure 2f, 98% of the EGFP-positive 4T1KO-1 cells, which also express ectopic MLK3, regain nuclear FRA-1 staining.

**Figure 3:**
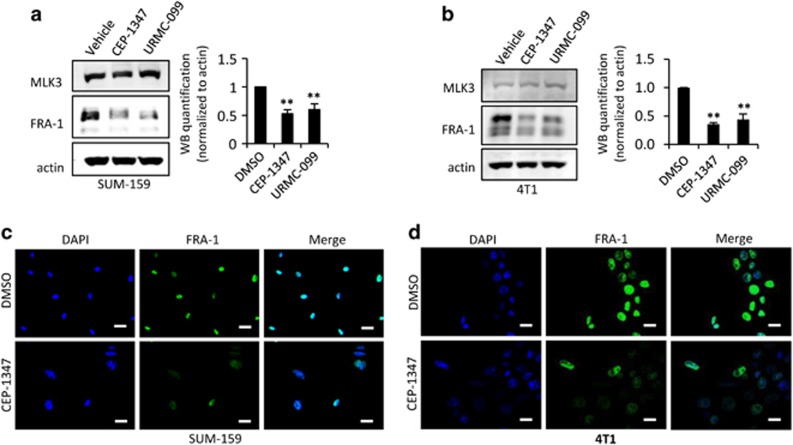
Pharmacological inhibition of MLKs reduces FRA-1 protein expression in TNBC lines. (**a**) SUM-159 cells and (**b**) 4T1 cells were treated with vehicle, 400 nm CEP-1347 or 400 nm URMC-099 for 24 h. Cellular lysates were subjected to immunoblotting with indicated antibodies. Western blot quantification of the indicated protein normalized to actin is expressed as mean±s.d. from at least three independent experiments. (**c**) SUM-159 cells and (**d**) 4T1 cells were seeded on coverslips, treated±400 nm CEP-1347 for 24 h, and subjected to immunofluorescence staining against FRA-1 antibody (green). Nuclei were counterstained with DAPI (blue); Scale bar, 25 μm in **c** and 50 μm in **d**.

To assess whether blockade of MLK activity reduces FRA-1 levels in highly invasive breast cancer cells, SUM-159 and 4T1 cells were treated with either CEP-1347 or URMC-099, MLK inhibitors with unrelated chemical structures, for 24 h and subjected to immunoblot analysis. As shown in Figure 3a and Figure 3b, treatment with either MLK inhibitor downregulates FRA-1 protein in both SUM-159 and 4T1 cells. Similarly, immunofluorescence staining of SUM-159 and 4T1 cells treated with CEP-1347 revealed loss of FRA-1 staining (Figure 3c and d), similar to the phenotype of the 4T1KO-1 cells. These findings further support a requirement for active MLK3 in FRA-1 expression in highly invasive breast cancer cell lines. Of note, MLK inhibitor treatment is sufficient to reduce both JNK and ERK signaling in 4T1 cells (Supplementary Figure 3). Further, overexpression, silencing or inhibition of MLK3 in breast cancer cells had no effect on levels of the related transcription factor c-FOS, reflecting specificity in MLK3 control of FRA-1 (Supplementary Figure 4).

### MLK3-activated JNK and ERK contribute to FRA-1 regulation

Our data show that active MLK3 induces expression of FRA-1. Ectopic expression of wild-type MLK3 in MCF7 cells increases both JNK and ERK activation, as judged by the levels of phospho-JNK and phospho-ERK. It is well established that MLK3 utilizes its catalytic activity to regulate JNK activity.^8, 9^ However, MLK3 can activate ERK either through either kinase-dependent signaling^26^ or kinase-independent scaffolding,^27^ depending upon context. As shown in Figure 3a, wild-type MLK3 increases both JNK and ERK activities and drives FRA-1 expression, but equivalent levels of the kinase dead mutant MLK3-K144M fail to upregulate FRA-1 and do not significantly increase JNK or ERK activity.

**Figure 4:**
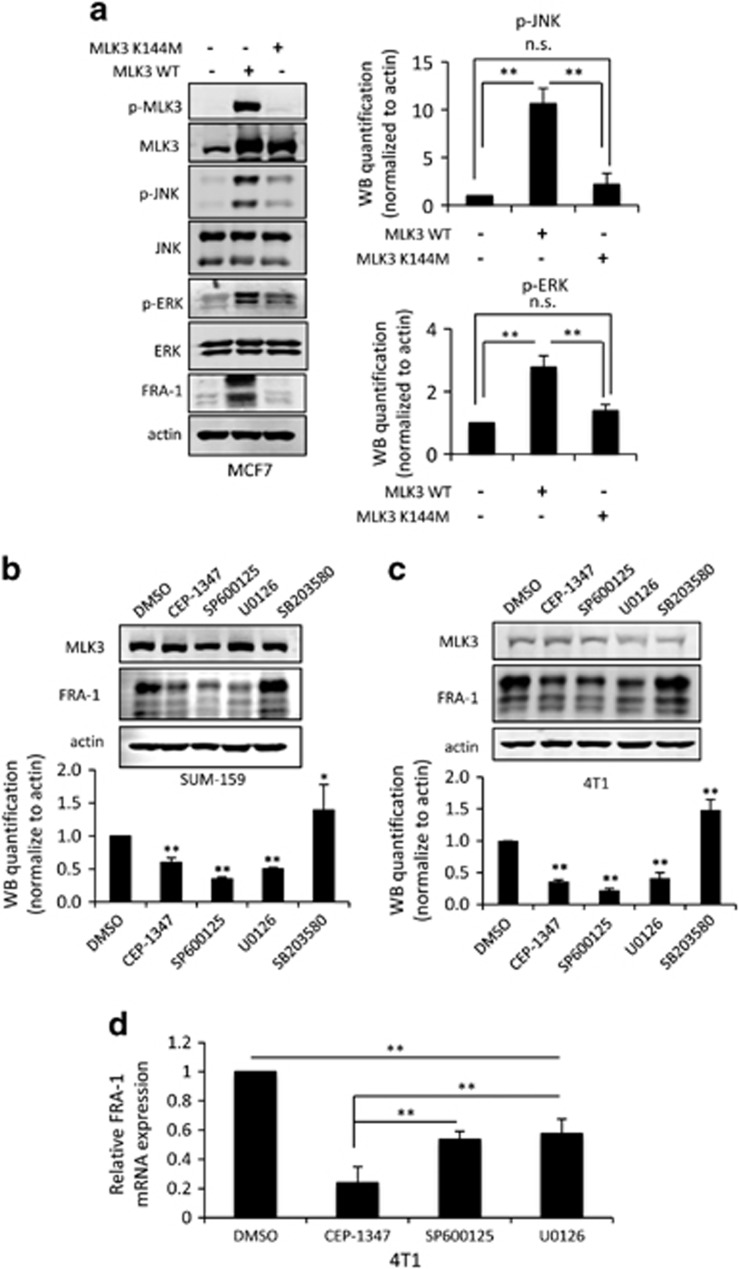
Both JNK and ERK pathways contribute to FRA-1 expression through MLK3 signaling. (**a**) MCF7 cells were transiently transfected with a wild-type MLK3 (pRK-MLK3) or a kinase dead MLK3 variant (pRK-MLK3-K144M) for 24 h, (**b**) SUM-159 and (**c**) 4T1 cells were treated with vehicle, 400 nm CEP-1347 (MLK inhibitor), 15 μm SP600125 (JNK inhibitor), 10 μm U0126 (MEK/ERK inhibitor) or 10 μm SB203580 (P38 inhibitor) for 24 h. Cellular lysates were subjected to immunoblotting with indicated antibodies. Western blot quantification of the indicated protein normalized to actin is expressed as mean±s.d. from at least three independent experiments. (**d**) The mRNAs from 4T1 cells treated with vehicle, 400 nm CEP-1347, 15 μm SP600125 and 10 μm U0126 for 24 h were subjected to qRT–PCR analysis with FRA-1 primers. Relative mRNA expression is displayed as mean±s.d. from at least three independent experiments performed in triplicate; NS, not statistically significant; **P<0.01.

To determine which MAPK signaling pathways are required for maintaining basal levels of endogenous FRA-1 in TNBC cells, cells were treated with small molecule inhibitors that block specific MAPK pathways, including SP600125 (JNK), U0126 (MEK/ERK) or SB203580 (P38), and the impact on FRA-1 protein levels was assessed. Treatment with the MLK, JNK or ERK inhibitor significantly decreased basal, endogenous FRA-1 levels in SUM-159 (Figure 4b) and 4T1 cells (Figure 4c). The P38 MAPK inhibitor SB203580 increased FRA-1 expression in both invasive breast cancer cell lines, perhaps reflecting the established antagonism between the JNK and p38 pathways.^28^ In a time course, treatment with either the JNK or MEK inhibitor resulted in a reduction of basal FRA-1 protein levels over time (Supplementary Figure 5), consistent with what has been observed on MEK inhibitor treatment of colon cancer cells.^29^ In this experiment, SP600125 effectively blocked JNK activity as judged by phospho-c-JUN levels, but had no impact on phospho-ERK. The MEK inhibitor, U0126, efficiently blocked ERK activation over the entire time course, although a slight diminution of phospho-c-JUN at later time points was observed, suggesting that inhibition of the ERK pathway may indirectly downregulate phospho-c-JUN, as has been shown in Ras-overexpressing thyroid cells.^30^ Treatment with either CEP-1347, SP600125 or U0126, for 24 h reduced FRA-1 transcript levels, as determined by qRT–PCR (Figure 4d), suggesting that both the JNK and ERK pathways contribute to FRA-1 transcriptional regulation.

### MLK3 increases MMP-1 and MMP-9 through FRA-1

**Figure 5:**
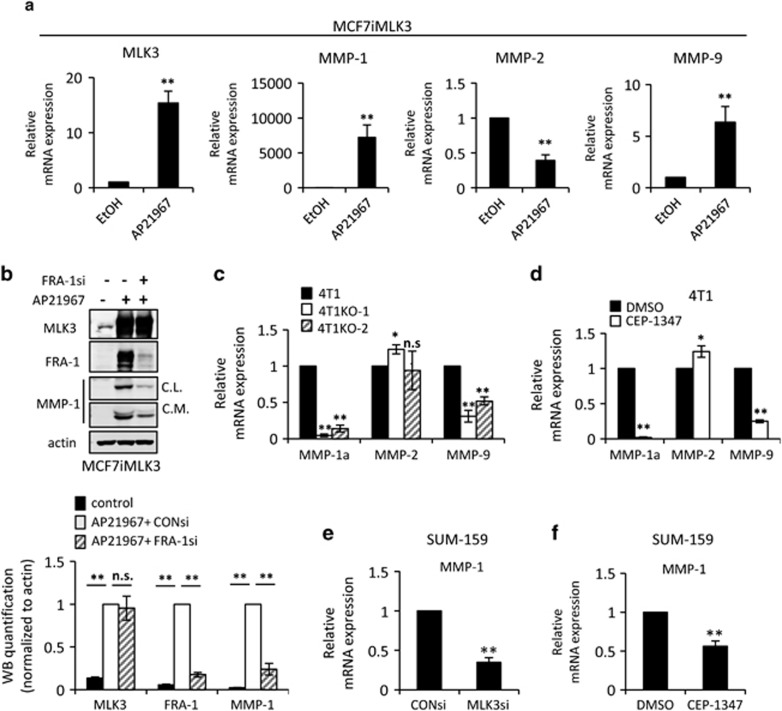
MLK3 induces MMP-1 and MMP-9 expression. Cellular lysates or mRNAs, as indicated, were isolated from (**a**) MCF7iMLK3 cells treated with vehicle or 50 nm AP21967 for 24 h, (**b**) MCF7iMLK3 cells treated with vehicle or with 50 nm AP21967 plus either 50 nm control or FRA-1 siRNA, as indicated, for 24 h, (**c**) parental 4T1 cells and two 4T1 MLK3-knockout clones (4T1KO-1 and 4T1KO-2), (**d**) 4T1 cells treated with vehicle or 400 nm CEP-1347 for 24 h, and SUM-159 cells treated with (**e**) 50 nm control or MLK3 siRNA for 24 h, or (**f**) vehicle or 400 nm CEP-1347 for 24 h. The mRNAs were subjected to qRT–PCR with primers to the indicated genes. Relative mRNA expression is displayed as the mean±s.d. from at least three independent experiments performed in triplicate. Cellular lysates were subjected to immunoblotting with indicated antibodies. Western blot quantification of the indicated protein normalized to actin is expressed as mean±s.d. from at least three independent experiments. Con, Control; CL, cellular lysate; CM, concentrated conditioned medium; NS, not statistically significant; *P<0.05; **P<0.01.

FRA-1 is an oncogenic member of the AP-1 transcription factor family,^31^ which regulates expression of genes involved in cancer progression, including MMPs. As MLK3 regulates both FRA-1 and cancer cell invasion, we hypothesized that it may control the expression of FRA-1-dependent MMPs, such as MMP-1,^15, 17, 32^ MMP-2^33, 34^ and MMP-9.^17^ As shown in Figure 5a, induced expression of MLK3 upregulates MMP-1 and MMP-9 mRNA but reduces MMP-2 mRNA levels. Both cytosolic and secreted MMP-1 protein can be detected on induction of MLK3 expression in MCF7iMLK3 cells, reflecting the strong upregulation of MMP-1 mRNA. Importantly, the increase in MMP-1 protein is abrogated by FRA-1 gene silencing (Figure 5b).

The mRNA levels of the same MMPs were evaluated in the 4T1 line and 4T1KO clones. By qRT–PCR analysis, MMP-1a, the functional ortholog of human MMP-1,^35^ is ablated in 4T1KO-1 and 4T1KO-2, compared with control parental 4T1 cells (Figure 5c). Loss of MLK3 decreases MMP-9 mRNA levels by 60 and 50% in 4T1KO-1 and 4T1KO-2 cells, respectively, compared with parental 4T1 cells. Similar effects on mRNA levels of MMPs were observed in 4T1 cells treated with CEP-1347 (Figure 5d). SUM-159 TNBC cells, which express MMP-1 but not MMP-9,^36^ show decreased MMP-1 mRNA levels upon MLK3 gene silencing or MLK inhibitor treatment (Figure 5e and f).

### MLK3 regulates cancer cell invasion and transendothelial migration

**Figure 6:**
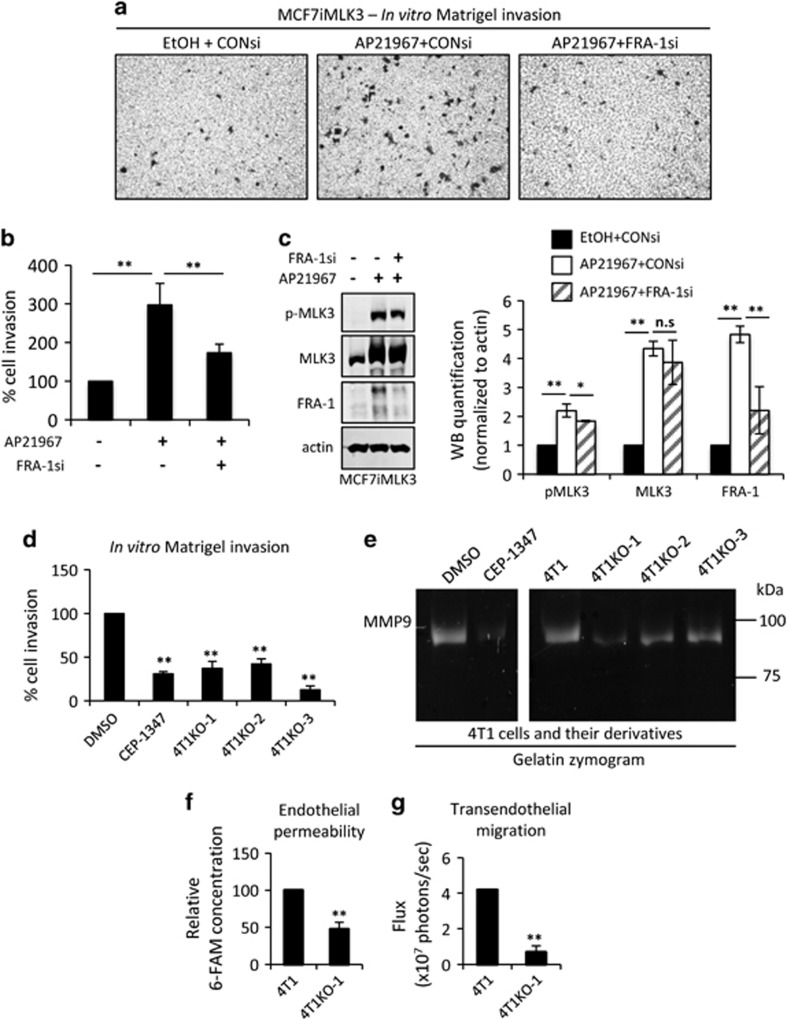
MLK3 regulates cancer cell invasion and transendothelial migration. (**a**–**c**) MCF7iMLK3 cells treated with vehicle or with 25 nm AP21967 plus either 50 nm control or FRA-1 siRNA, as indicated, and (**d**) parental 4T1 cells treated with vehicle or 400 nm CEP-1347 and MLK3-KO 4T1 clones (KO-1, KO-2 and KO-3) were subjected to an *in vitro* Matrigel transwell invasion assay for 24 h. Relative cell invasion, with control set at 100%, is expressed as mean±s.d. from three independent experiments. (**e**) A representative image from three independent experiments of gelatin zymography of conditioned medium from parental 4T1 cells treated with vehicle or 400 nm CEP-1347 and MLK3-KO 4T1 clones (KO-1, KO-2 and KO-3). (**f** and **g**) Parental 4T1 and 4T1KO-1 were subjected to transendothelial migration toward 10% FBS. Endothelial permeability was assessed using 5(6)-Carboxyfluorescein (6-FAM)-conjugated albumin, and transendothelial migration was assessed using bioluminescence imaging as described in the 'Materials and Methods' section. Relative 6-FAM conjugated albumin concentration and bioluminescence activity are expressed mean±s.d. from three independent experiments performed in triplicate. **P<0.01. *P<0.1. NS, not significant.

To investigate the requirement of FRA-1 in MLK3-driven cancer cell invasion, the impact of FRA-1 silencing on Matrigel transwell invasion of MCF7iMLK3 cells was determined. As shown in Figure 6a–c, induced expression of MLK3 in MCF7iMLK3 cells upregulates FRA-1 protein levels and enhances invasion. Silencing of FRA-1 reduces MLK3-induced invasion, indicating that FRA-1 functions downstream of MLK3 in this context. Of note, we previously found a role for MLK3 in JNK-mediated phosphorylation of paxillin in MCF10A and TNBC cells.^8^ In MCF7 cells, we observe a modest increase in phospho-paxillin on MLK3 expression, which is unaffected by FRA-1-silencing (Supplementary Figure 6) suggesting that MLK3 regulation of paxillin is independent of FRA-1, and consistent with the idea that MLK3 controls multiple pathways in cancer cell migration and invasion.

In TNBC models, tumor cell-derived MMP-9 is required for Matrigel invasion and for formation of pulmonary metastases.^37, 38, 39^ MMP-1 is crucial for transendothelial migration and vascular intravasation.^40^ As MLK3 deletion decreases both MMP-1 and MMP-9 levels, we tested whether deletion of MLK3 impacts Matrigel invasion and transendothelial migration in TNBC cells. Indeed three independent 4T1 MLK3 KO clones show impaired invasion through Matrigel compared with parental 4T1 cells. Treatment with CEP-1347 similarly inhibits Matrigel invasion (Figure 6d) of 4T1 cells. We then performed gelatin zymography to evaluate levels of secreted MMP-9. Notably, MMP-9 can be distinguished from the other major gelatinase MMP-2, based on molecular weights of 92 kDa and 72 kDa, respectively. Gelatin zymography of conditioned medium from 4T1 cells shows a single predominant band corresponding to MMP-9; and secreted MMP-9 levels are reduced in conditioned media from 4T1 cells treated with CEP-1347 or from multiple 4T1 MLK3 KO clones (Figure 6e).

Transendothelial migration assays were performed to assess the ability of 4T1 or 4T1KO-1 cells to disrupt and invade through a confluent endothelial cell monolayer. Migration of luciferase-expressing cancer cells through the endothelial cell monolayer was quantified by bioluminescence imaging. The 4T1KO-1 clone was chosen for these experiments because its bioluminescence activity is comparable to that of parental 4T1 cells (Supplementary Figure 7). Parental 4T1 cells increase permeability of the endothelial cell layer as measured by leakage of fluorescently labeled albumin from the upper into the lower transwell chamber, whereas 4T1KO-1 cells show markedly reduced endothelial permeability (Figure 6f). Compared with parental 4T1 cells, 4T1KO-1 cells show a fourfold reduction in transendothelial migration (Figure 6g). These data indicate that MLK3 contributes to cancer cell-induced endothelial permeability and is required for transendothelial migration.

### FRA-1 and MMP-1a are upregulated in circulating tumor cells derived from TNBC tumors

**Figure 7:**
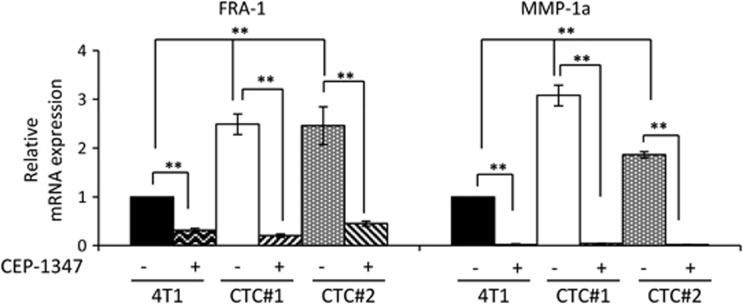
FRA-1 and MMP-1a are upregulated in 4T1-derived circulating tumor cells. The mRNAs isolated from parental 4T1 cells and from circulating tumor cell lines, 4T1-CTC#1 and 4T1-CTC#2, treated with vehicle or 400 nm CEP-1347 for 24 h were subjected to qRT–PCR analysis using FRA-1- and MMP-1a-specific primers. Relative mRNA expression is displayed as mean±s.d. from at least three independent experiments performed in triplicate; **P<0.01.

The established role of MMP-1 in cancer intravasation, coupled with our findings that MLK3 controls MMP-1 levels, prompted us to evaluate the components of the MLK3–FRA-1–MMP-1 signaling axis in CTCs. A clonogenic assay was used to isolate CTCs from the blood of mice bearing 4T1-luc2 mammary tumors and associated metastases. By phase contrast imaging, isolated CTC lines (4T1-CTC) show distinct morphology compared with the morphology of the parental 4T1 cells. Many of these cells are able to detach and re-attach to tissue culture plates (Supplementary Figure 8). Bioluminescence imaging demonstrated that the isolated CTC lines retain luciferase activity, confirming their origin from the 4T1-luc2 tumors (Supplementary Figure 8). Both 4T1-CTC lines show increased FRA-1 and MMP-1a mRNA expression, compared with parental 4T1 cells. Furthermore in the 4T1-CTC lines, FRA-1 and MMP-1a levels are dependent upon MLK activity, as their levels are decreased by CEP-1347 treatment (Figure 7). Co-expression and mutual exclusivity analysis (Supplementary Figure 9) revealed a statistically significant tendency towards co-occurrence of MLK3 and FRA-1 gene expression, as well as MMP-1 and FRA-1.

## Discussion

Metastasis is overwhelmingly the cause of breast cancer-related death, yet the complexity of the metastatic process makes it therapeutically challenging to treat.^41^ Previous studies in our lab and others have demonstrated that MLK3 is crucial for TNBC metastasis.^7, 8^ Herein, we utilized the CRISPR/Cas9 approach to deplete MLK3 in the highly aggressive 4T1 mammary cancer model to elucidate the role(s) of MLK3 in discrete steps of metastasis and to identify the key signaling pathways through which MLK3 regulates these events. Mechanistically, we have deciphered a novel function for MLK3 in controlling FRA-1 in breast cancer cells. In this context, both JNK and ERK signal downstream of MLK3 (Figure 4, Supplementary Figure 3) to enhance FRA-1 expression. FRA-1 regulation is complex. JNK is well known to phosphorylate c-JUN,^42^ the AP-1 member, which is required for transcription of FRA-1.^43^ ERK, in turn, phosphorylates FRA-1, which enhances the stability of the FRA-1/c-JUN heterodimer.^14, 29^ Thus MLK3 signaling is well poised to regulate FRA-1/c-JUN-mediated transcription.

FRA-1 has emerged as a key driver of metastatic progression in multiple cancer types including breast cancer,^11, 14, 15, 17^ lung cancer,^18^ colorectal cancer^16, 44, 45^ and glioblastoma.^46^ FRA-1 not only controls expression of genes involved in cell motility, epithelial-to-mesenchymal transition^13, 45, 47, 48^ and cell invasion,^15, 17, 32, 49^ but it also controls proliferation,^15, 50, 51^ metastatic outgrowth^44^ and the stem cell phenotype of cancer cells.^52^ Several FRA-1-regulated genes^15, 17, 32, 49^ have been demonstrated to facilitate cancer cell invasion including MMP-1, MMP-2 and MMP-9.^17, 18^ Zhan *et al.*^53^ have previously reported that, in ovarian cancer cells, MLK3 is important for expression of MMP-1, -2, -9 and -12 and is required for ovarian cancer invasion. Our studies show that in TNBC models, MLK3 deletion decreases both MMP-1 and MMP-9 expression (Figure 5) but does not significantly affect MMP-2 levels. Functionally, MMP-9 facilitates extracellular matrix remodeling and basement membrane degradation and, like MLK3, is critical for TNBC metastasis.^37^ Likewise, MMP-9 silencing in multiple TNBC lines has been shown to block Matrigel invasion,^37^ analogous to our findings that MLK3 deletion or CEP-1347 treatment inhibits Matrigel invasion (Figure 6).

In TNBC, hematogenous metastasis, which requires transendothelial migration^54^ is more common than lymphatic spread.^55^ During vascular intravasation and extravasation, cancer cells must disrupt endothelial barrier integrity and transmigrate through the endothelial layer. MMP-1 is an interstitial collagenase-I required for fibrillar collagen remodeling.^56, 57, 58^ However non-collagenolytic mechanisms of MMP-1 are implicated in transendothelial migration of tumor cells.^40^ In epidermoid cancer, for instance, tumor cell-derived MMP-1 increases endothelial barrier permeability by proteolytically activating the endothelial thrombin receptor PAR-1 and facilitating transendothelial migration.^40^ In our study, TNBC cells induce endothelial barrier permeability and transmigrate through an endothelial barrier, whereas, MLK3-deleted tumor cells fail to induce permeability and their transendothelial migration is impaired (Figure 6). These findings suggest that one mechanism through which MLK3 facilitates TNBC metastasis could be through vascular intravasation and, possibly, extravasation.

CTCs have risen to prominence as potential prognostic and predictive biomarkers for metastatic burden, metastatic recurrence and therapeutic response.^59, 60, 61, 62, 63, 64, 65, 66, 67, 68, 69, 70^ Our finding that 4T1-CTCs have elevated mRNA levels of FRA-1 and MMP-1a compared with parental 4T1 cells (Figure 7) is important in light of several lines of evidence supporting a key role for MMP-1 in CTCs, epithelial-to-mesenchymal transition and metastatic progression. Furthermore, MMP-1 was identified as a key gene upregulated in infiltratative self-seeding CTCs.^71^ Elevated MMP-1 was also observed in TNBC MDA-MB-231 subclones selected for their ability to metastasize to lung^72^ and brain.^73^ Recently, single cell gene expression analysis studies utilizing TNBC patient-derived xenograft models showed that MMP-1, as well as components of a proliferative gene signature, was significantly increased in late stage, high burden metastatic cells compared with early stage, low burden metastatic cells.^74^ These data, along with the role of MMP-1 in vascular intravasation, suggest that MMP-1 is not only required for early stages of metastatic process, but may also contribute to colonization.

TNBC is considered the most aggressive subtype of breast cancer; however, therapeutic options are limited. A major challenge is to identify important targetable signaling pathways in TNBC. MMP-1 expression is significantly elevated in aggressive breast tumors and correlates with both tumor size and grade^75^ pointing to MMP-1 as a promising therapeutic target. Indeed, MMPs, including MMP-1, have a long history as targets for cancer therapeutics yet early clinical trials using MMP inhibitors were unsuccessful due, in part, to inadequate preclinical and clinical design, lack of drug specificity and high toxicity.^76, 77^ Recently FRA-1, an upstream regulator of MMP-1, has also emerged as a key driver of cancer progression; however, transcription factors are not readily druggable. In one study, an existing inhibitor of the FRA-1 regulated gene, adenosine receptor A_2B_, ADORA2B, was shown to block formation of lung metastases in a TNBC experimental metastasis xenograft model.^19^ However, it is unclear whether targeting a single FRA-1-regulated gene will always be sufficient to halt breast cancer progression, as FRA-1 controls a suite of genes involved in cancer invasion and metastasis.^45^ Based on our findings, we propose that an alternative strategy would be to target MLK3, an upstream regulator of FRA-1.

Multiple MLK inhibitors exist, including CEP-1347^78^ and URMC-099.^79^ Our data show that both of these MLK inhibitors, built upon different chemical scaffolds, reduce FRA-1 levels in 4T1 cells. In addition, either CEP-1347 treatment or MLK3 deletion reduces FRA-1, MMP-1, and MMP-9 expression to similar levels in multiple TNBC cells, indicating that MLK3, specifically, controls FRA-1, MMP-1 and MMP-9 expression. CEP-1347 progressed through Phase II/III clinical trials for Parkinson’s disease, and although it failed to delay progression, no significant toxicity was observed,^80^ suggesting that it could potentially be repurposed for breast cancer treatment.

In summary, we provide evidence that MLK3 signaling is a crucial regulator of FRA-1 and its target genes, MMP-1 and MMP-9 in models of TNBC. As a consequence, depletion or inhibition of MLK3 in TNBC cells impairs both Matrigel invasion and transendothelial migration. Consistent with these findings, FRA-1 and MMP-1 are upregulated in isolated CTC lines from a TNBC model. Importantly the MLK inhibitor, CEP-1347, which blocks invasion, also reduces FRA-1 and MMP-1 in CTC lines. Taken together, our data reveal important roles of MLK3 during basement membrane degradation and transendothelial migration and suggest that MLK3 inhibitors may be a useful addition to the limited armament for combating TNBC.

## Materials and methods

### Chemicals and antibodies

#### Chemicals

5(6)-Carboxyfluorescein (6-FAM), bovine serum albumin, gelatin and 4′,6-diamidino-2-phenylindole (DAPI) were from Sigma-Aldrich (St Louis, MO, USA). SP600125, U0126 and SB203580 were from Calbiochem (San Diego, CA, USA). CEP-1347 and CEP-11004 were generously provided by Cephalon, Inc., a wholly owned subsidiary of Teva Pharmaceuticals, Ltd (North Wales, PA, USA). AP21967 was provided by Ariad Pharmaceuticals (Cambridge, MA, USA). Calcein AM and Simple Blue SafeStain were from Invitrogen (Carlsbad, CA, USA).

#### Antibodies

Anti-MLK3 (A-20) (for detection of murine MLK3), anti-FRA-1 (R-20), anti-JNK1/3 (C-17), anti-ERK1 (K-23), anti-P38 (C-20), anti-actin (C-2), anti-p-c-JUN (S63)(KM-1) and anti-c-JUN (H-79) were from Santa Cruz Biotechnology (Santa Cruz, CA, USA). Rabbit anti-MLK3 (C-terminal) (for detecting human MLK3) was from Epitomics (Cambridge, MA, USA). Anti-p-MLK3, anti-p-ERK-1/2 (T202/Y204)(E-10), anti-p-JNK1/2 (T183/Y185)(81E11) and anti-p-P38 (T180/Y182) (#9216) were obtained from Cell Signaling (Danvers, MA, USA). Anti-MMP-1 (#36665 R) was purchased from R&D systems (Minneapolis, MN, USA), anti-p-paxillin S178 (#A300-100 A) was purchased from Bethyl Laboratory (Montgomery, TX, USA). IRDye 800CW goat anti-mouse IgG, IRDye 680 goat anti-rabbit IgG and IRDye 800CW donkey anti-goat IgG were from Li-COR Biosciences (Lincoln, NE, USA). Goat anti-rabbit IgG conjugated with Alexa Fluor 488 and 546 was from Invitrogen and used for immunofluorescence staining.

### Cell lines

MCF7, obtained from ATCC (Manassas, VA, USA), have been recently authenticated and were maintained in DMEM supplemented with 10% fetal bovine serum (FBS). MCF7iMLK3 cells engineered to inducibly express MLK3 were previously described.^9, 22^ ZR-75-1 (from ATCC) and 4T1-luc2 (Perkin Elmer, Waltham, MA, USA) cells were maintained in RPMI-1640 (Gibco, Life Technology, Grand Island, NY, USA) with 10% FBS. SUM-159-GFP cells (a gift from Dr Chengfeng Yang (University of Kentucky)) were maintained in Ham’s F-12 (Gibco) supplemented with 5% FBS, 5 μg/ml insulin, 1 μg/ml hydrocortisone and containing penicillin/streptomycin. The cell lines were routinely tested for mycoplasma contamination.

### RNA interference and plasmid transfection

For siRNAs, Mission siRNA Universal Negative control #1, siRNA duplexes targeting human MLK3 (5′-CUGACUGCCACUCAUGGUG-3′ and its antisense)^9, 81^ and human FRA-1 (5′-GGGCAGUGACGUCUGGAG-3′ and its antisense)^15^ were from Sigma-Aldrich. Lipofectamine 2000 (Invitrogen) was used as a transfection reagent.

Plasmids, pRK-MLK3 or pRK-MLK3 K144M, were previously described.^23^ Lipofectamine 2000 and Lipofectamine 3000 were used to transfect MCF7 and ZR-75-1 cells, respectively. In a recovery assay of MLK3-knockout 4T1 cells, pCMV-EGFP-MLK3^82^ expression vector was reverse-transfected into 4T1KO-1 cells using Lipofectamine 3000.

### CRISPR-Cas9n constructs

The MLK3 CRISPR construct was generated based on a previously described protocol.^83^ Briefly, two pairs of guide RNAs for a CRISPR-Cas9n construct were designed (crispr.mit.edu) to target exon 1 of murine MLK3 (Supplementary Figure 2) and were cloned into pSpCas9n(BB)-2A-GFP (PX461)^83^ (a gift from Dr Feng Zhang; Addgene plasmid #48140). After reverse transfection using Lipofectamine 3000, GFP-positive clones were screened for MLK3 deletion by immunoblotting. Genomic DNAs from selected clones were collected and amplified using forward primer 5′-ATGGAGCCCTTGAAGAACCT-3′ and reverse primer 5′-ACGGTAGACCTTGCCGAAG-3′. Purified PCR products were subjected to TOPO TA cloning (Invitrogen). At least five clones were subjected to nucleotide sequencing to identify the genomic alterations.

### Immunoblot analysis

Cellular lysates were prepared in lysis buffer (1% NP-40, 150 mm sodium chloride and 50 mm Tris, pH 8.0) and immunoblotting was performed as described.^8, 9^

### Immunofluorescence analysis

Immunofluorescence staining was performed as previously described.^8^ Images were acquired and, if indicated, quantified from *N*>100 cells per group, using an Olympus fluorescence microscope and MetaMorph software.

### Gelatin zymography

4T1 cells or their derivatives (2.5 × 10^5^) were seeded to 35 mm culture dishes. The following day, the cells were incubated in serum-free medium for 24 h, in the presence of CEP-1347, as indicated. Conditioned media corresponding to equal cellular equivalents were loaded onto and run through 10% polyacrylamide gels containing 1 mg/ml gelatin. The gels were incubated for 1 h in 2.5% Triton X-100, developed for 24 h at 37 °C in 50 mm Tris-HCl buffer, pH 7.6, containing 5 mm CaCl_2_ and 200 mm NaCl, and finally stained for 16 h with Simple Blue SafeStain (Invitrogen). After 30 min destaining with water, gels were scanned and the images were processed using Image J software.

### Quantitative real-time PCR

Total RNAs were extracted using the RNeasy kit (Qiagen, Valencia, CA, USA) and cDNA synthesis was performed using a cDNA reverse transcription kit (Applied Biosystems, Foster City, CA, USA). Real-time qPCR was performed using either PerfeCTa SyBR green superMix (Quanta, Gaithersburg, MD, USA) or SYBR Green Master mix (Applied Biosystems). Specific primer sequences were designed using PrimerBank^84^ (see Supplementary Table 1 for full list of primers).

### *In vitro* Matrigel invasion assay

*In vitro* Matrigel invasion assay was performed as previously described.^8, 9^ Briefly, MCF7iMLK3 cells were transfected with 50 nm Control siRNA or MLK3 siRNA as described above for 16 h, and then serum-deprived for an additional 8 h in the presence of vehicle or 25 nm AP21957. The cells (1 × 10^5^) were seeded into the upper chamber of 5 μm Matrigel-coated transwell chamber and allowed to invade in the presence of vehicle or 25 nm AP21967 for 24 h. For 4T1 cells and their derivatives, cells were serum-deprived overnight and 2 × 10^4^ cells introduced into the upper chamber and allowed to invade for 24 h toward 10% FBS in the presence of indicated inhibitors. Mitomycin C (2 μg/ml) was included to eliminate possible effects of cell proliferation. Experiments performed in duplicate were repeated at least three times.

### Transendothelial migration and endothelial permeability

Transendothelial migration was assessed essentially as described.^40, 85^ Briefly, 1 × 10^5^ EA.hy926 cells were grown as a confluent monolayer on 5 μm pore transwell inserts for 1–2 days and 1 × 10^5^ 4T1 cells, or their derivatives, that had been deprived of serum for 18 h were introduced to the upper chamber. 4T1 cells were allowed to migrate toward 10% FBS for 24 h. To measure permeability of the endothelial layer, 5(6)-Carboxyfluorescein (6-FAM)-conjugated albumin (10 μm final concentration) was added to the upper chamber and culture medium was collected from the bottom chamber after 30 min at 37 ^o^C. Permeability was determined by measuring the fluorescence due to leakage of 6-FAM-conjugated albumin (excitation=495 nm, emission=520 nm) into the bottom chamber. To measure transendothelial migration, the cells inside the transwell inserts were wiped out with the cotton swabs, and the extent of migration through the transwell membranes was then determined by relative bioluminescence activity.

### CTC isolation from the 4T1 tumor bearing mice

This experiment was carried out in accordance with standard protocols approved by All University Committee on Animal Use and Care at Michigan State University. Briefly, puromycin-resistant 4T1-luc2 cells (7.5 × 10^5^ cells) were injected into the fourth mammary gland of 8-week-old female athymic nu/nu mice (*N*=2). After 24 days, the two mice were killed and CTCs were isolated as previously described.^86^ Briefly, 200 μl blood collected by cardiac puncture was cultured in RPMI-1640 supplemented with 20% FBS, 2 μg/ml puromycin, and penicillin/streptomycin for 10 days. Approximately 40 colonies were obtained from each blood sample, pooled and propagated as populations, named 4T1-CTC#1 and 4T1-CTC#2.

### Statistical analysis

Results are expressed as mean±standard deviation (s.d.). An unpaired, two-tailed Student’s *t*-test was used to calculate the *P*-value, and *P*<0.05 is considered statistically significant. At least three independent experiments were performed unless otherwise noted.
