# Supplementary material for: MLK3 regulates FRA-1 and MMPs to drive invasion and transendothelial migration in triple-negative breast cancer cells
Source: Oncogenesis. 2017 Jun 12;6(6):e345–. doi: 10.1038/oncsis.2017.44 (PMC5519193; doi:10.1038/oncsis.2017.44)
Supplement: Supplementary Information [file oncsis201744x1.pdf]

Supplementary Table 1. List of primers

	Forward primer	Tm	Reverse Primer	Tm
hMLK3	GCAGCCCATTGAGAGTGAC	60.5	CACTGCCCTTAGAGAAGGTGG	61.8
hFRA-1	CAGGCGGAGACTGACAAACTG	62.9	TCCTTCCGGGATTTTGCAGAT	61.5
hMMP-1	GAGCAAACACATCTGAGGTACAGGA	58.5	TTGTCCCGATGATCTCCCCTGACA	61.5
hMMP-2	CCCACTGCGGTTTTCTCGAAT	62.9	CAAAGGGGTATCCATCGCCAT	62
hMMP-9	AGACCTGGGCAGATTCCAAAC	62	CGGCAAGTCTTCCGAGTAGT	61.3
hGAPDH	GGCTGAGAACGGGAAGCTTGTCAT	61.4	AGCCTTCTCCATGGTGGTGAAGA	60.8
mFRA-1	ATGTACCGAGACTACGGGGAA	61.8	CTGCTGCTGTCGATGCTTG	61.5
mMMP1a	AACTACATTTAGGGGAGAGGTGT	60.2	GCAGCGTCAAGTTTAACTGGAA	61.3
mMMP-2	CAAGTTCCCCGGCGATGTC	63	TTCTGGTCAAGGTCACCTGTC	61.3
mMMP-9	GGACCCGAAGCGGACATTG	63	CGTCGTCGAAATGGGCATCT	62.6
mGAPDH	TGTGTCCGTCGTGGATCTGA	58.1	CCTGCTTCACCACCTTCTTGA	57.2

(A)

(B)

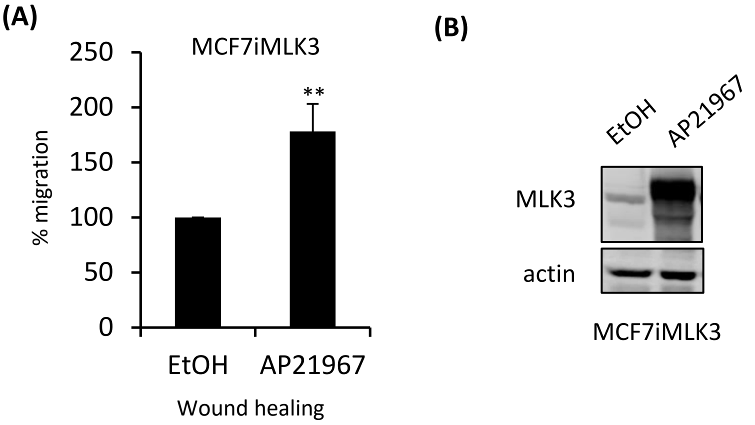

Supplementary Figure 1. Induced MLK3 expression drives wound closure in MCF7iMLK3 cells. MCF7iMLK3 (4x10^{5}) cells were seeded in 35-mm dishes to obtain a confluent monolayer on the following day. The wounds were then generated using p200 tip and the cells were allowed to migrate for 24 h in the presence of vehicle or 25 nM AP21967. Mitomycin C (2 µg/mL) was added to exclude the effect of cell proliferation. **(A)** % migration of control and AP21967-treated MCF7iMLK3 cells which is calculated by the relative migratory ability ((Width of the wound at t= 0) – (width of the wound at t = 24 h)/(width of the wound at t = 0) compared with control group. % migration is expressed as mean ± SD from three independent experiments; ** $p < 0.01$. **(B)** Cellular lysates of MCF7iMLK3 were collected at the end of the wound healing experiments and were subjected to immunoblotting with indicated antibodies.

(A)

	**Guide A**	**Guide B**
**M1**	CATTGGGCTCATAGTCGAACAGG	CCTGAGGAAGGGCGACCGTGTGG
**M4**	ATTGGGCTCATAGTCGAACAGGG	GGCAGGACGAACTAGCCCTGAGG

(B) **M1 clones**

Guide A → ← Guide B

parental **CCTGTTCGACTATGAGCCCAATG**GGCAGGACGAACTAGC**CCTGAGGAAGGGCGACCGTGTGG**

4T1KO-1_allele 1 **CCTGTTCGACTATGAGCCCAATG**GGCAGGACGAACTAGC**CCTGAGGAA*GGGCGACC*GGGCGACCGTGTGG**

4T1KO-1_allele 2 **CCTGTTCGACTATGAGCCCAATG**GGCAGGACGAACTAGC**CCTGA------------------CCGTGTGG**

M4 clones

Guide A → ← Guide B

parental **CCCTGTTCGACTATGAGCCCAATGGGCAGGACGAACTAGCCCTGAGGAAGGGCGACCGTGTGG**

WT **CCCTGTTCGACTATGAGCCCAATGGGCAGGACGAACTAGCCCTGAGGAAGGGCGACCGTGTGG**

4T1KO-2 **CCCTGTTCGACTATGAGCCCAATGGG-------------------------CCTGAGGAAGGGCGACCGTGTGG**

4T1KO-3_allele 1 **CCCTGTTCGACTATGAGCC-------------------------------A-----------A---AAGGGCGACCGTGTGG**

4T1KO-3_allele 2* **---**

(C)

FRA-1

GFP/
DAPI

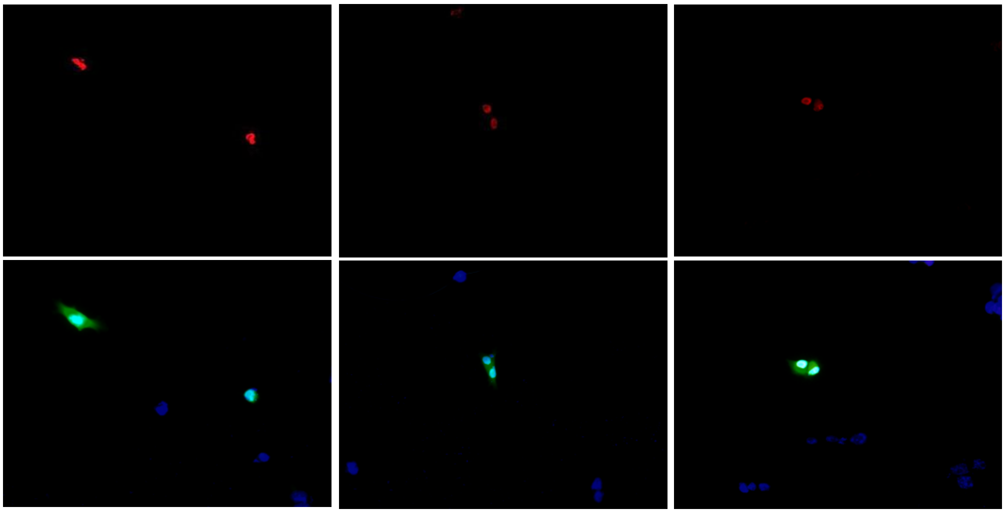

Supplementary Figure 2. Genome-edited sequencing of 4T1 CRISPR clones. (A) Two pairs (M1 and M4) of guide RNAs targeting exon 1 region of murine MAP3K11 genomic DNA were designed using crispr.mit.edu. **(B)** Genomic DNAs from parental 4T1 cells, a WT clone and three 4T1 MLK3 KO clones: 4T1KO-1, 4T1KO-2, and 4T1KO-3 were isolated and subjected to sequencing to identify genomic alteration. The PAM sequences are underlined. **(C)** The bi-cistronic vector pCMS-EGFP-MLK3 was introduced into 4T1KO-1 cells via reversed transfection for 24-28 h, and the cells were subjected to immunofluorescence staining with FRA-1 antibodies (red). DAPI, shown in blue, was used as a marker for nuclei. This experiment was repeated three times.

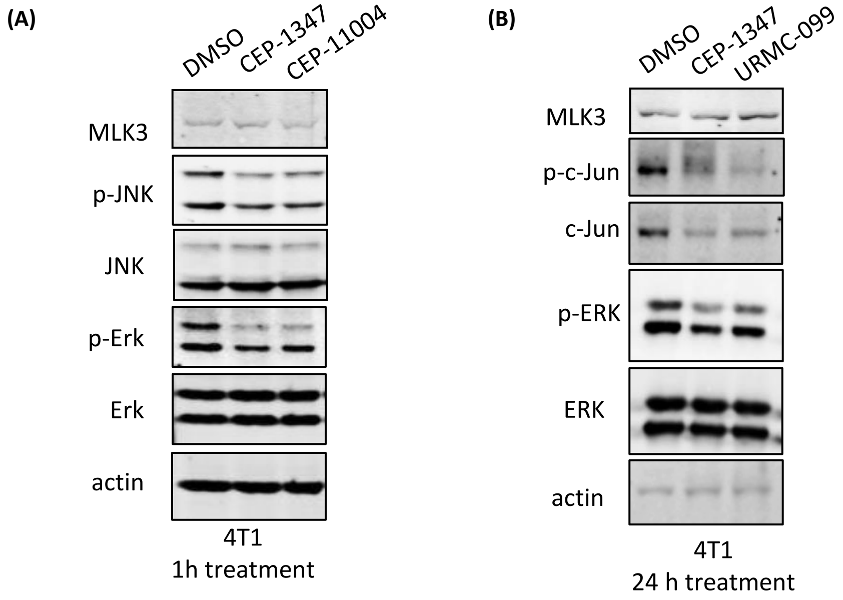

Supplementary Figure 3. MLK inhibitor treatment downregulates ERK and JNK activities. 4T1 cells were treated with **(A)** DMSO, 400 nM CEP-1347 or 400 nM CEP-11004 for 1 h.**(B)** 4T1 cells were treated with DMSO, 400 nM CEP-1347 or 400 nM URMC-099 for 24 h. Cellular lysates were subjected to immunoblotting with indicated antibodies. All experiments were repeated at least three times.

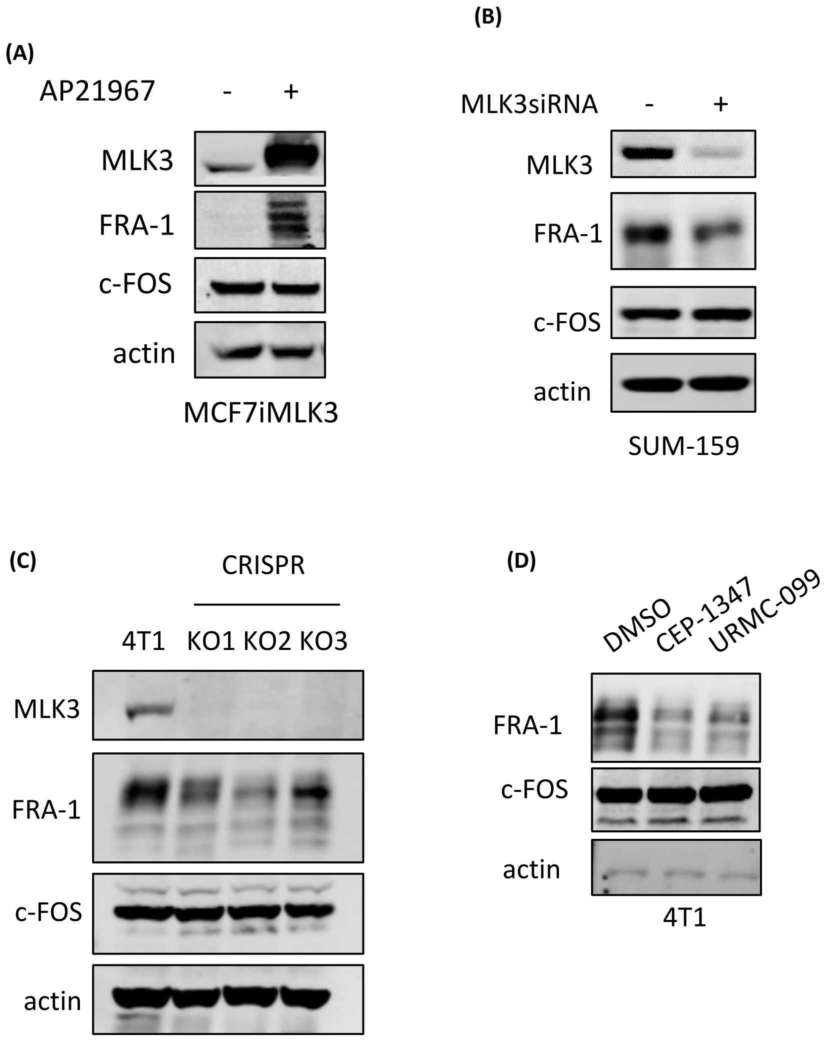

Supplementary Figure 4. MLK3 regulates FRA-1 but not c-FOS expression in breast cancer cells Cellular lysates were collected from **(A)** MCF7iMLK3 cells treated with vehicle or 25 nM AP21967 for 24 h, **(B)** SUM-159 treated with 50 nM controlsiRNA or MLK3siRNA for 24 h, **(C)** 4T1 cells and their derivatives, **(D)** 4T1 cells treated with 400 nM CEP-1347 or 400 nM URMC-099. All lysates were subjected to immunoblotting with indicated antibodies and were repeated at least three times.

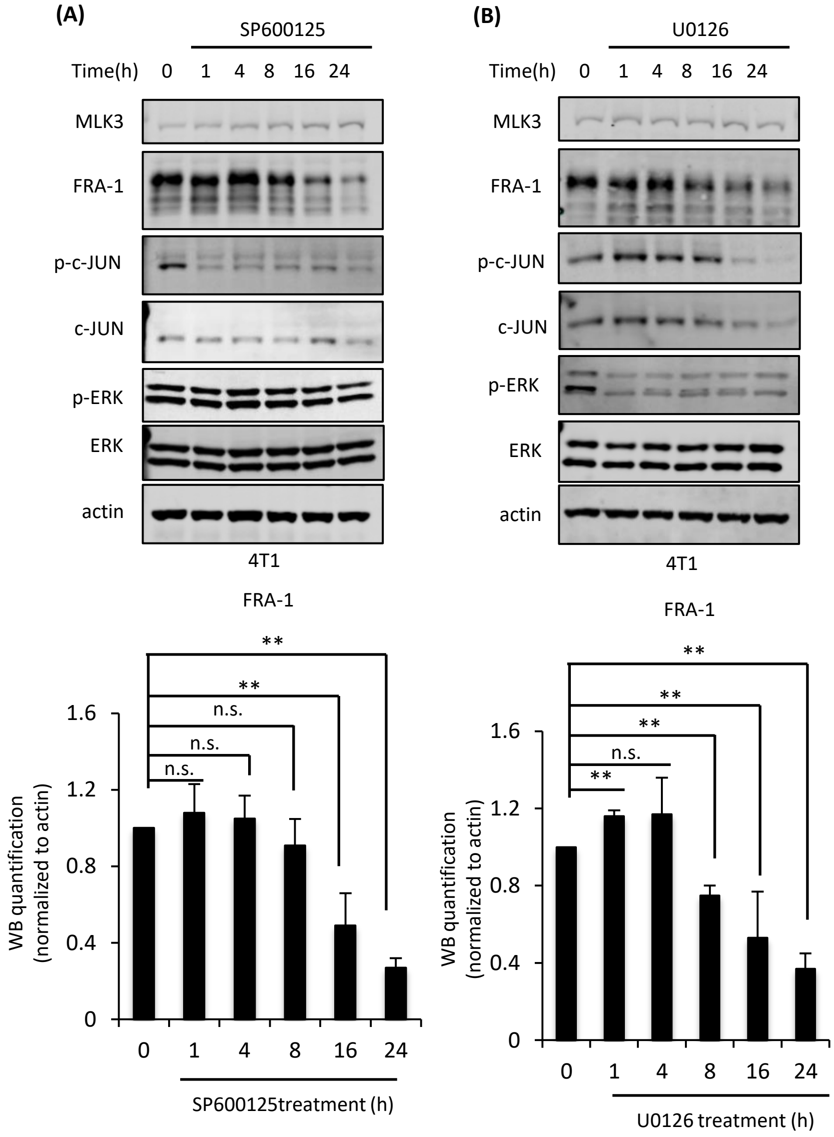

Supplementary Figure 5. Time course analyses for JNK and ERK pathway inhibition on FRA-1 expression. 4T1 cells were treated with **(A)** 15 µM SP600125 (JNK inhibitor) and **(B)** 10 µM U0126 (MEK/ERK inhibitor) for 1, 4, 8, 16, 24 h. Cellular lysates were subjected to immunoblotting with indicated antibodies. Western blot quantification of the indicated protein normalized to actin is expressed as mean ± SD from at least three independent experiments; n.s. – not statistically significant; ** $p < 0.01$.

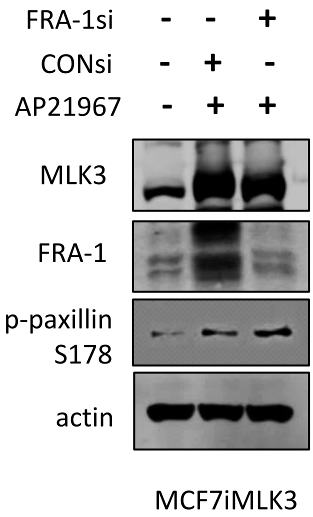

Supplementary Figure 6. Loss of FRA-1 does not affect MLK3-induced phosphorylation of paxillin. MCF7iMLK3 cells were treated with vehicle control, 25 nM AP21967 + 50 nM controlsiRNA or 25 nM AP21967 + 50 nM FRA-1siRNA for 48 h. Cellular lysates were collected and subjected to immunoblotting with indicated antibodies. All experiments were repeated at least three times.

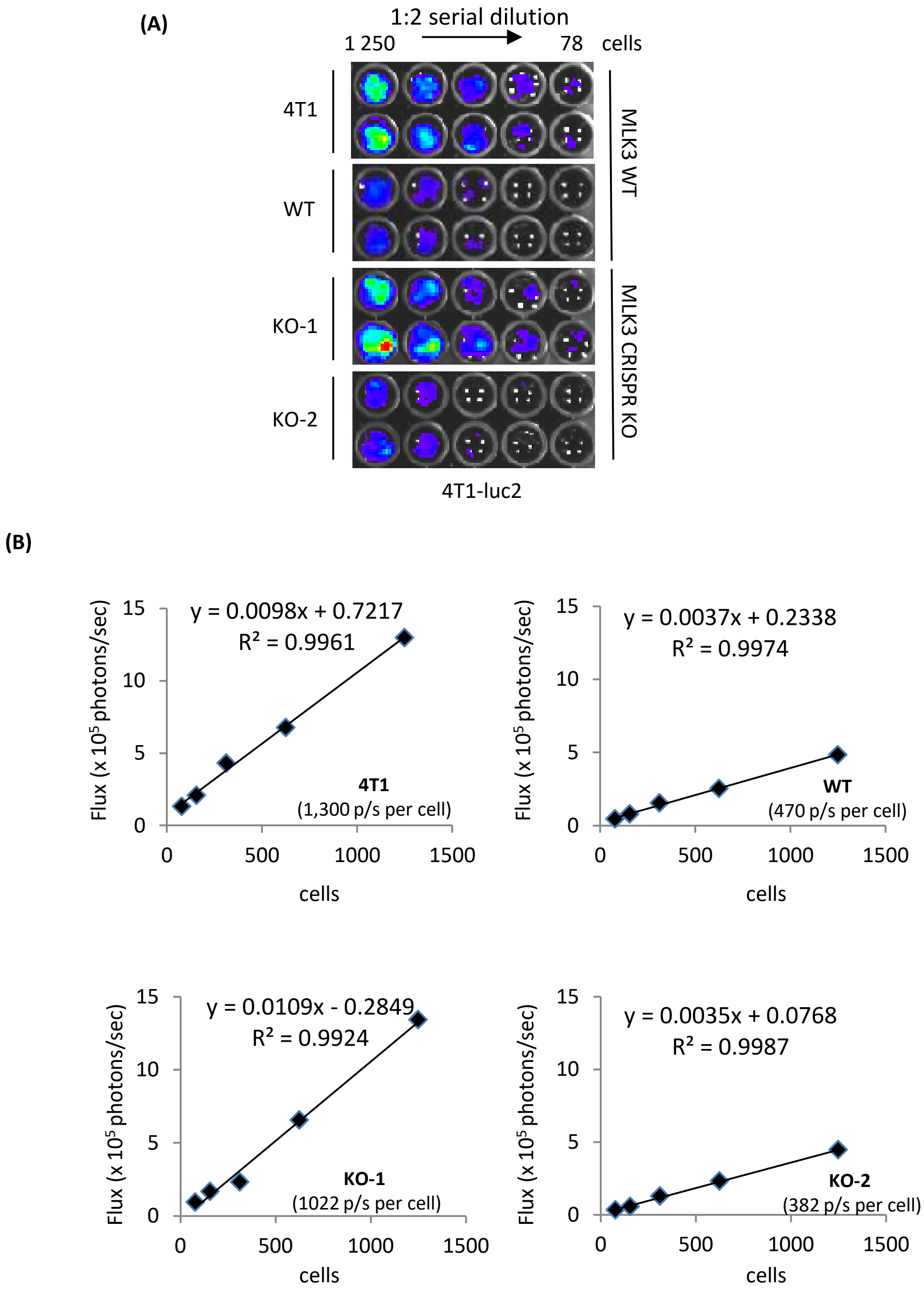

Supplementary Figure 7. Bioluminescence of 4T1, WT, and 4T1 MLK3 KO clones. (A) Parental 4T1-luc2 cells, a WT clone and two independent 4T1 MLK3 KO clones (4T1KO-1 and 4T1KO-2) were seeded in a black, 96-well plate overnight. Cells were then treated with 2 µg/mL D-luciferin for 3 min and the bioluminescence was measured using the IVIS system. **(B)** Standard curve generated from **(A).**The bioluminescence activity was plotted as a function of cell number to determine the average photon flux/s of each cell line .

(A)

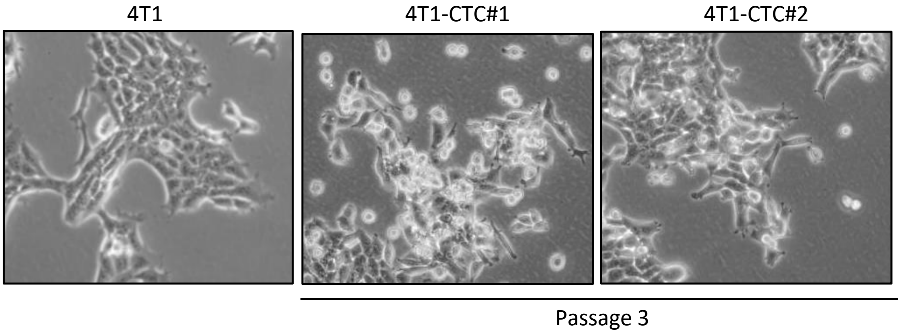

(B)

Samples	Number of CTC colonies* at Day 9
CTCs from mouse#1 + puro	42
CTCs from mouse#2+ puro	30

*From 200 ul blood collected from cardiac puncture.

(C)

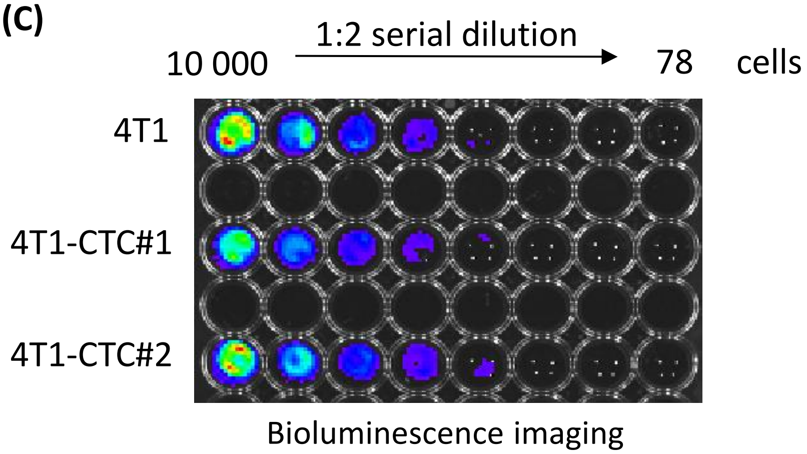

Supplementary Figure 8. 4T1-CTC cells are the derivative of 4T1-luc2 cells. (A) Representative images of parental 4T1 and 4T1-CTC lines isolated from the blood of 4T1 (containing luc2 construct) tumor-bearing mice using a colony formation assay. **(B)** Number of colonies from blood cultures in the presence of puromycin (2 µg/mL) were count at day 9. Colonies from each plate were pooled and named 4T1-CTC#1 and 4T1-CTC#2. **(C)** Parental 4T1-luc2**,** 4T1-CTC#1 and 4T1-CTC#2 cells were seeded in a black, 96-well plate overnight. Cells were then treated with 2 µg/mL D-luciferin for 3 min and bioluminescence was measured using the IVIS system.

(A)

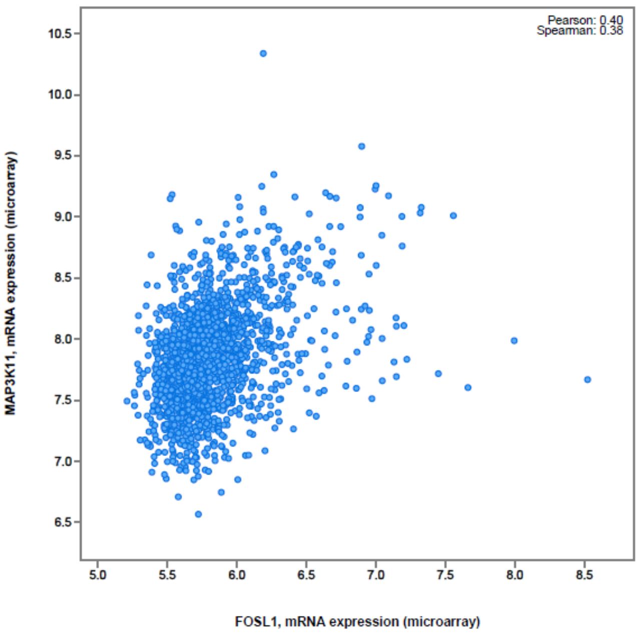

(B)

	Gene A	Gene B	p-value	Log Odds Ratio	Association
METABRIC (n=2509)	MAP3K11	FOSL1	4.51E-13	2.21	Tendency towards co-occurrence
	MAP3K11	MMP1	5.89E-06	1.326	Tendency towards co-occurrence
	FOSL1	MMP1	7.29E-05	1.439	Tendency towards co-occurrence

	Gene A	Gene B	p-value	Log Odds Ratio	Association
TCGA (n= 1105)	MAP3K11	FOSL1	0.008	1.573	Tendency towards co-occurrence
	FOSL1	MMP1	0.004	2.016	Tendency towards co-occurrence

Supplementary Figure 9. Co-expression of MLK3, FRA-1, and MMP-1 in breast tumors (A) mRNA Co-expression of MAP3K11 and FOSL1 in METABRIC data. **(B)** Co-occurrence analysis of MAP3K11, FOSL1, MMP1, MMP9 in METABRIC and TCGA data. Co-occurrences that reach statistical significance, along with their p-values and Log Odds Ratios, are shown. cBioPortal http://www.cbioportal.org/ (Curtis et al., Nature, 2012; Pereira et al., Nat Comm, 2016; TCGA).
